# THE ROLE OF WORK AND RETIREMENT IN COGNITIVE AND BRAIN AGING

**DOI:** 10.1093/geroni/igz038.090

**Published:** 2019-11-08

**Authors:** Gizem Hueluer, George W Rebok

**Affiliations:** 1 University of Zurich, Zurich, Switzerland, Switzerland; 2 Johns Hopkins Bloomberg School of Public Health, Baltimore, Maryland, United States

## Abstract

According to the “use it or lose it” hypothesis of cognitive aging, cognitive enrichment and cognitively engaging activities are associated with the maintenance of high levels of cognitive functioning in old age. Similar ideas have been brought forward with respect to characteristics of individuals’ work environment, with more cognitively enriching work demands providing an optimal environment for cognitive development and maintenance. The goal of this research group is to showcase new developments in research on work, retirement and cognitive aging. Hülür et al. examine the role of perceived work environment for cohort differences in trajectories of cognitive change based on 56-year longitudinal data from the Seattle Longitudinal Study. Andel et al. use data from the Swedish Adoption/Twin Study of Aging to examine trajectories of cognitive aging before vs. after retirement with two-slope growth curve models. Zulka et al. conduct a systematic literature review on the association between retirement and cognition and examine the role of factors such as occupational experiences and the cognitive domain studied. Burzynska et al. investigate the relationship between stressful and stimulating occupational exposures and structural brain health and cognition in older age. The discussion by George Rebok will focus on how these findings contribute to our understanding of the role of occupational experiences for cognitive and brain aging and how they can be utilized to promote maintenance of cognitive functioning in old age.

